# The use of combined fenestrated and bifurcated endografts in fenestrated aortic repair

**DOI:** 10.1186/s42155-025-00579-2

**Published:** 2025-08-07

**Authors:** Eric Dorenberg, Anne-Marte Schrøder-Aasen, Beate Lindberg, Rune Andersen, Steinar Guvåg, Ulrik Carling

**Affiliations:** 1 Department of Radiology, Section of Interventional Radiology, Oslo University Hospital, Oslo, Norway; 2 Faculty of Medicine, University of Oslo, Oslo, Norway; 3 Department of Cardiovascular, Thoracic and Vascular Surgery, Section of Vascular Surgery, Oslo University Hospital, Oslo, Norway

**Keywords:** Aortic aneurysm, Thoracoabdominal aortic aneurysm, Fenestrated endovascular aneurysm repair, Endovascular technique, Endovascular aortic repair

## Abstract

**Background:**

The aim of this study was to compare the use of combined fenestrated and bifurcated aortic endografts to the standard modular design including a proximal fenestrated and a distal, bifurcated endograft. The combined design allows for a modification of the procedure that may contribute to lowering the risk of damaging the target vessel stents and reducing the perioperative obstruction of the ipsilateral access vessel.

**Methods:**

Consecutive patients treated with fenestrated aortic repair between December 2020 and December 2022 were included in this retrospective, single center study. Technical success was analyzed, including the integrity of the target vessel (TV) stents assessed on perioperative CT. Further, the duration during which the large introducer had to be kept in the access vessel was analyzed. Finally, we report technical data on the endograft design, adverse events and midterm results.

**Results:**

Twelve patients were treated with a modular endograft (group A) and 13 patients with a combined endograft design (group B). Technical success was 100% in both groups, however there were 4 deformed target vessel stents in group A, none in group B. The duration of potential flow reduction due to a large introducer in the access vessel was significantly shorter in group B than group A (median 54 min vs. 109.5 min, *p* < 0.05). No adverse events were reported in any of the groups. The observation period was shorter in group B (median 18 months vs. 33 months, *p* < 0.05). Except for one case of aneurysm growth in group A, all other patients in both groups showed stable or decreased aneurysm size without TV occlusions.

**Conclusions:**

The integration of the bifurcation on the fenestrated endograft may contribute to the prevention of damage of the TV stents and has potential to reduce the duration of perioperative limb obstruction.

## Background

**Fig. 1:**
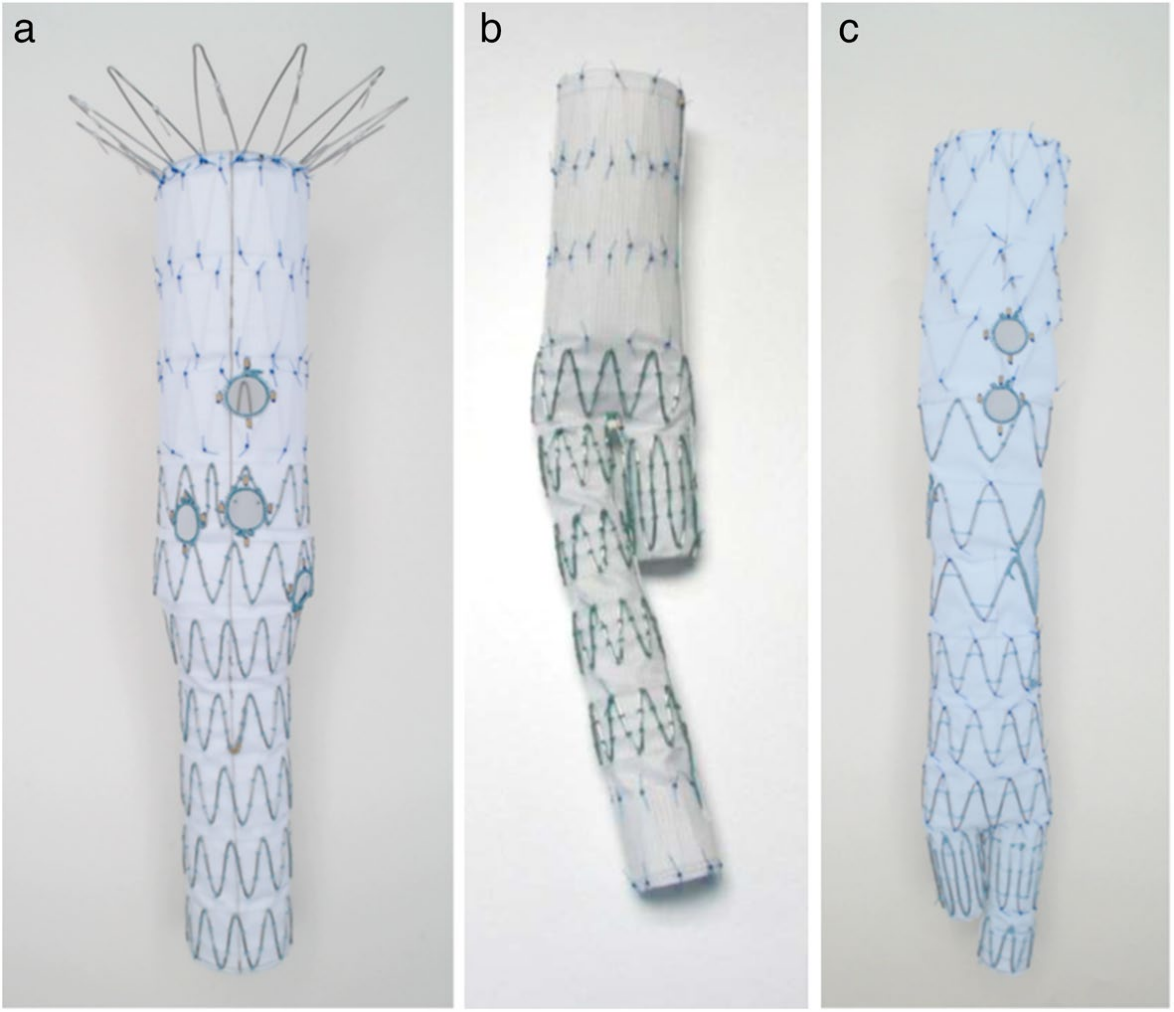
Images showing the difference in stentgraft design: modular design with a tubular, fenestrated graft (**a**) that has to be extended with a bifurcated distal component (**b**) as opposed to a combined fenestrated and bifurcated design (**c**). Images by courtesy of COOK Medical

During the last decade, fenestrated endovascular aortic repair (FEVAR) has gained acceptance for the treatment of juxtarenal and thoracoabdominal aortic aneurysms. Cohort studies have reported short- and mid-term results comparable to open repair and the technique is now part of international guidelines [1–3]. Most studies refer to the COOK Zenith fenestrated endograft (COOK Medical, Bloomington, IN, USA), which routinely is planned in a modular design. It consists of a fenestrated, tubular component and a separate distal bifurcated endograft that usually is extended bilaterally with standard iliac limbs (Fig. 1a and b). The bifurcated endograft is placed after the visceral and renal arteries (target vessels; TV) have been stented through the fenestrations. The dilator tip of the bifurcated graft usually passes the level of the TV stents, which protrude into the aortic lumen. This implies the risk of deformation of the TV stents with the consequence of type 3 endoleaks.

As an alternative to the modular design, COOK Medical can manufacture grafts in which the bifurcation is integrated in the fenestrated component resulting in a combined fenestrated and bifurcated graft (Fig. 1c), which then only has to be extended with iliac legs. This design results in increased graft length that might make precise placement more difficult. Further, the contralateral limb limits the space for catheterization and stenting of the fenestrations. However, the design allows for a modified order of the procedural steps which may contribute to lowering the risk of TV stent deformation. Further, this modified procedure may shorten the time during which a large introducer is placed in the femoral artery and thus reduce the obstruction of flow to the ipsilateral extremity.

As our institution during the years 2021 and 2022 gradually shifted from using fenestrated endografts with separate bifurcations to combined endografts, we aimed to compare the technical success, the effects on TV stent deformations and the potential reduction in obstruction time of the access vessel in these two groups.

## Material and methods

### Patients

For this retrospective study, all patients that had undergone a FEVAR procedure between December 2020 and December 2022 using a device from COOK Medical were included. We identified 12 patients treated with a modular endograft design (group A) and 13 patients who received a fenestrated endograft with integrated bifurcation (group B). The study was approved by the hospital review board, with waiver of written informed consent.

### Procedure

**Fig. 2:**
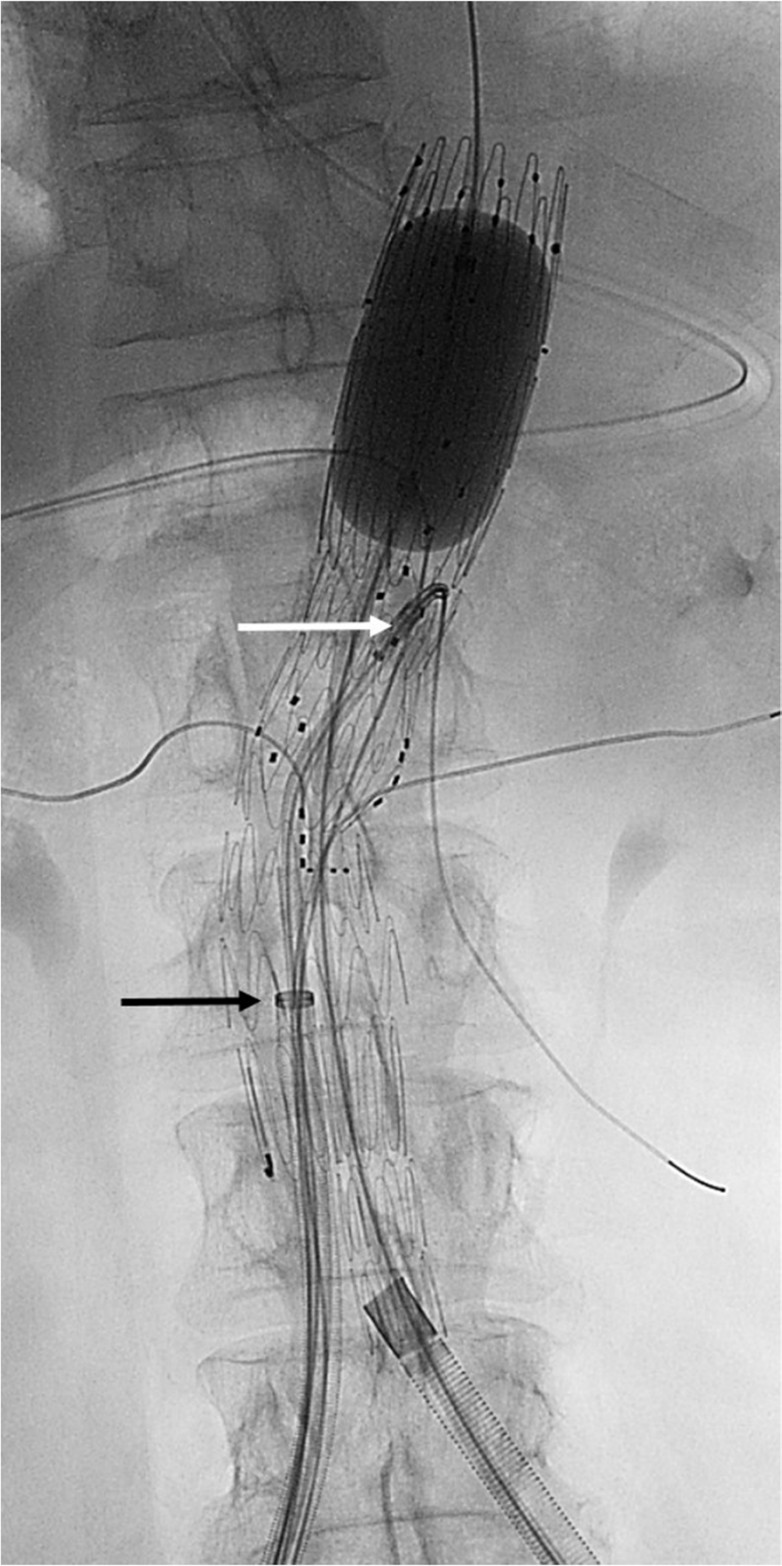
Screenshot taken during a procedure with a combined fenestrated and bifurcated endograft. The 14 French introducer (black arrow) from the contralateral side is advanced through the contralateral gate. Guidewires are positioned through the fenestrations to the visceral and renal arteries. During inflation of a balloon (Coda, COOK Medical, Bloomington, IN, USA) in the proximal landing zone, the steerable sheath is kept in the superior mesenteric artery (white arrow)

Planning of the endografts was in all cases made by the same interventional radiologist in collaboration with the London-based custom-made device planning center from COOK Medical, based on pre-operative computed tomography angiography (CTA). Main endograft access was preferably planned from the left side in order to optimize ergonomics for catheterization of the fenestrations. The choice of endograft design was gradually shifted from a modular design to combined graft design during the period, without specific anatomical or technical criteria for choosing one over the other. All procedures were performed under general anesthesia by members of the same team of two interventional radiologists and two vascular surgeons. Since the introduction of fEVAR at our institution in 2015, the same team had performed more than 100 fEVAR procedures in addition to numerous abdominal and thoracic aortic repairs before the inclusion period of this study. The hybrid operating room was equipped with an Artis Zeego (Siemens, Erlangen, Germany) allowing for intraoperative image fusion guidance and a sliding CT-scanner (Siemens SOMATOM Edge, Siemens Healthcare GmbH, Erlangen, Germany). Based on the quality and dimension of the common femoral artery, ipsilateral access was obtained by either open or percutaneous approach. Depending on the dimension of the contralateral iliac leg endograft, a 14 Fr or 16 Fr Dry Seal® introducer (Gore Medical, Newark, DE, USA) was placed percutaneously on the contralateral side. In cases with open access, a shunt was created by placing an antegrade 5 Fr introducer in the ipsilateral superficial femoral artery and connecting it to the contralateral introducer. After deployment of the fenestrated endograft, the contralateral introducer was advanced into the main body. Using a 7 Fr or 8.5 Fr steerable introducer (Tour Guide, Medtronic. Minneapolis, MN, US or Heart Span, Merit Medical, Jordan, UT, USA), guidewires (Storq, Cordis, Miami Laker, FL, USA or Rosen, COOK Medical, Bloomington, IN, USA) were placed through the fenestrations to the visceral and renal arteries. The steerable introducer was kept in the superior mesenteric artery during the release of the diameter reducing ties and balloon dilation of the proximal graft landing zone (Fig. 2). In group A the TVs were stented before placing the bifurcated graft and the iliac legs. In group B the endograft design allowed for a modification of the procedural steps: after the placement of the endograft, the fenestrations are catheterized, and wires are placed in the target vessels from the contralateral side. Before stenting the TVs, the main sheath can be removed and after iliac extension, the ipsilateral access is closed.

### Study parameters

Technical success was defined as successful deployment of all endograft components and placement of covered stents through the fenestrations to the visceral and renal arteries. Perioperative CT was performed in all patients as previously described [4]. The integrity of the TV stents was assessed by DSA and intraoperative CT in all patients. Further analysis included how long the large introducer of the fenestrated graft had to be kept in the access vessel. All procedures were documented by frequent image storage allowing for assessment of time intervals between different steps of the procedures. The time point of the introduction of the main graft to the femoral artery was registered in all patients (T1). In patients treated with a separate bifurcation, we defined the time point when stenting of the last TV was completed as T2. In patients treated with a combined endograft, we defined the time point of the balloon inflation in the landing zones of the endograft after catheterization of all TVs as T3. Since T2 and T3 describe the last step of the procedure before the large introducer can be removed from the ipsilateral groin, we used the time interval of T1-T2 for group A and T1-T3 for group B as surrogate markers for how long the large introducer had to be kept in the access vessel.

Finally, we also examined potential adverse events and analyzed mid-term results as assessed on follow-up CTA.

### Statistics

Data are presented as median and range, or numbers and percentages. Mann–Whitney-U tests in IBM SPSS 29.0 (IBM Corp., Armonk, NY, USA) were used for comparison of age, aneurysm diameters, follow-up period and time of introducer in the access vessel between the two groups. A *p*-value > 0.05 was considered statistically significant.

## Results

### Patients and endografts

The vast majority of patients were males in both group A and B (92.6% and 91.7%), the median age at the procedure was 74 years in both groups (range 59–82 in group A, 63–83 in group B). Most patients had juxtarenal aneurysms (69.2% in group A, 83.3% in group B) with a median diameter of 58 mm (range 45–68) and 60 mm (range 50–69), respectively. Three patients in group A had earlier been treated with either EVAR (*N* = 2) or open aortic repair.

(*N* = 1), while one patient in group B had undergone open repair. All of the 12 endografts in group A had 4 fenestrations. The majority of endografts in group B had 4 fenestrations (*N* = 11), the two remaining had 3 fenestrations and either one branch (*N* = 1) or a proximal scallop (*N* = 1). The combined fenestrated and bifurcated endografts in group B were significantly longer than the fenestrated, tubular endografts in group A with a median length of 203 mm (range 139–248) and 176.5 mm (range 159–217), respectively (*p* = 0.005).

### Technical findings

All procedures were technically successful in both groups. An antegrade shunt was used in 8 cases in both groups. Perioperative imaging after placement of all components revealed deformation with type 3 endoleak in 4 renal stents in group A. All of these were repaired by either additional balloon dilatation or stent placement during the same procedure. There were no findings related to TV stents in group B. The time interval during which the main body introducer had to be kept in the access vessel was 109.5 min (range 77–183) in group A and 54 min (range 37–162) in group B (*p* < 0.001).

### Clinical findings

No adverse events, especially no complications related to perioperative limb ischemia were registered.

One patient in group B died unrelated to aortic disease before follow-up. Median follow-up with CTA was longer in group A than group B (36 months, range 13–54 and 18, range 4–30, respectively, *p* = 0.024). There was one case of aneurysm sac increase in group A, all other follow-up imaging showed decreased or stable sac size in both groups. There were no TV occlusions observed during the follow-up period.

## Discussion

In this study comparing patients treated with FEVAR using either a “traditional” modular design or a combined fenestrated and bifurcated endograft, we found 4 cases of renal stent deformation in the first group and none in the second group. Target vessel stents being compressed are not an uncommon finding and reported in 7% of cases described by Tenorio et al. in 2020 [5]. Since the stent deformation often occurs during passing of the TV stent level with the dilator tip of distal endograft, attempts have been made to design short tip delivery systems, but even using such in the distal bifurcated component, Karelis et al. [6] found 6 cases (26%) of TV stent compression or inadequate flaring. Three of the cases were in fenestrations that had not been crossed by the dilator tip of the bifurcated component, but by the dilator tip of the iliac legs. Using a combined endograft design and a modified procedure sequence as described above, the TV stents are not crossed at all, or only with the contralateral iliac leg delivery systems, which usually are either 12 Fr or 14 Fr. This might contribute to the absence of TV stent findings in our material Table 1.

**Table 1:** Combined fenestrated and bifurcated aortic endografts. Overview over the study groups and main results. Data are presented as number (%), or median (range)

	Group A modular endograft *N* = 12	Group B Combined fenestrated and bifurcated endograft *N* = 13	*p*-value
Age (years)	74 (59–82)	74 (63–83)	1.00
Technical success	12 (100)	13 (100)	
TV-stent findings	4 (33)	0	
Large introducer in access vessel (minutes)	109.5 (77–183)	54 (37–162)	.001
Months to last follow-up	36 (13–54)	18 (4–43)	.024
Aneurysm diameter at last follow-up			
Decrease	7 (58)	7 (54)	
Stable	4 (33)	5 (38)	
Increase	1 (8)	0	
missing		1 (8)	

Two other papers describing the use of combined fenestrated and bifurcated endografts reported excellent technical success and 100% TV patency during the follow-up of up to one year [7, 8]. As opposed to our population, these papers mainly focused on patients that had undergone standard EVAR. The main reason for using this endograft design in these patients was to avoid the short overlap between a fenestrated tube and a short bifurcation with inverted limb. Despite the short distance between the graft bifurcation and the renal arteries, the authors reported no difficulties in catheterization of the target vessels. This is in accordance with our experience. The combined fenestrated and bifurcated endografts used in our material are longer than the components in a modular design. However, our limited experience did not reveal any difficulties in precise positioning. The fact that most of our patients were males who tend to have larger access vessels than females, might contribute to this experience.

Complex endovascular aortic repair involves the use of large introducer systems, which may compromise perfusion of the lower limb during instrumentation. In an experimental study, Jonsson et al. found metabolic changes due to lower limb ischemia in uncomplicated EVAR cases [9]. Complications from prolonged limb ischemia include the need for fasciotomy as reported in large series [10]. Strategies like establishing shunts have been published by Hanley et al. [11] and adopted in our center after the unpublished experience of complicated limb ischemia in two cases. According to the usual treatment sequence, after balloon dilation of the proximal anastomosis of the fenestrated component, the TV stent are placed and flared before inserting the distal bifurcated graft. The ipsilateral iliac leg is usually delivered through the introducer of the bifurcated graft; thus a large 20–22 Fr sheath is kept in the ipsilateral access vessel throughout most of the operating time. Even without considering the obstruction time due to the insertion of the bifurcated graft, in our limited experience the time during which the large introducer of the fenestrated graft has to stay in the femoral artery could be reduced from median 109 min to less than one hour by using combined grafts and a modified procedure sequence. This time reduction may omit the need of a perioperative shunt and reduce the risk of ischemic complications especially in patients with narrow iliac and femoral arteries. In neither group did we experience any complications related to access vessel occlusion, as an ipsilateral shunt readily was used. With more experience using the combined endograft design, we now usually use percutaneous access and omit the shunt.

The use of a modular design has been recommended in order to reduce the force applied on the visceral segment with fenestration in case of distal migration of the bifurcated part distally [6, 12]. Concerns have been raised that migration of a combined graft would lead to compression and occlusion of the TV stents. However, during the limited observation time in our and other studies, this complication has not been reported [7, 8].

Obviously, the small sample size in our study is a major limitation. In the setting of highly individualized endografts, it is not likely that a randomized trial comparing the use of a modular solution to combined fenestrated and bifurcated endografts will be conducted, but larger case series or a multicenter registry would allow for more robust conclusions. In addition, the observation period for patient groups differs significantly. This obviously limits the results of the comparison of mid-term outcomes, but has, however, no influence on the periprocedural results.

## Conclusions

Combined fenestrated and bifurcated endografts can be placed with excellent technical success and may reduce the risk of TV stent deformation. Further, they allow for a modified procedure that can reduce the time of flow obstruction to the ipsilateral limb significantly. Larger studies comparing our findings to the use of the standard, modular systems and investigating further potential benefits, and longer follow-up are warranted.

## Data Availability

The dataset used for this retrospective study are available as an anonymised Excel-file on reasonable request.

## Abbreviations

CTA: Computed tomography angiography
EVAR: Endovascular aortic repair
FEVAR: Fenestrated endovascular aortic repair
TV: Target vessel
